# Development and Validation of an HPLC Method for Determination of Amifostine and/or Its Metabolite (WR-1065) In Human Plasma Using OPA Derivatization and UV Detection

**Published:** 2015

**Authors:** Nasim Samiei, Seyed Mohsen Foroutan, Alireza Shafaati, Afshin Zarghi

**Affiliations:** a *Department of Pharmaceutics, School of Pharmacy, Shahid Beheshti University of Medical Sciences, Tehran, Iran. *; b *Department of Pharmaceutical Chemistry, School of Pharmacy, Shahid Beheshti University of Medical Sciences, Tehran, Iran.*

**Keywords:** Amifostine, WR-1065, human plasma, HPLC, OPA derivatization

## Abstract

A rapid, sensitive and reproducible HPLC method was developed and validated for the analysis of amifostine (AMF) and/or its metabolite, WR-1065 in human plasma. The method involves the alkylation of free sulfydryl group with iodoacetic acid followed by derivatization of the drug and its metabolite with *o*-phthaldialdehyde (OPA) and UVdetection at 340 nm. The derivatized AMF and WR-1065 were eluted in less than 11 min, and in the case of the metabolite with no interferences from the endogenous plasma peaks. Cystein was used as the internal standard. Analysis was carried out on a Eurosphere Performance (RP-18e, 100 × 4.6 mm) analytical column. The mobile phase was a mixture of methanol and phosphate buffer 0.03 M pH = 2.7 at a ratio of 40: 60v/v, respectively, with a flow rate of 1.5 mLmin^-1^. Limit of detection was 0.5 µgmL^-1^. The method involved a simple extraction procedure for AMF and/or its metabolite and analytical recovery was 90 ± 0.9%.The calibration curve was linear over the concentration range of 1-200 µgmL^-1^. The coefficients of variation for intra-day and inter-day assays were less than 10%.

## Introduction

**Figure 1:**
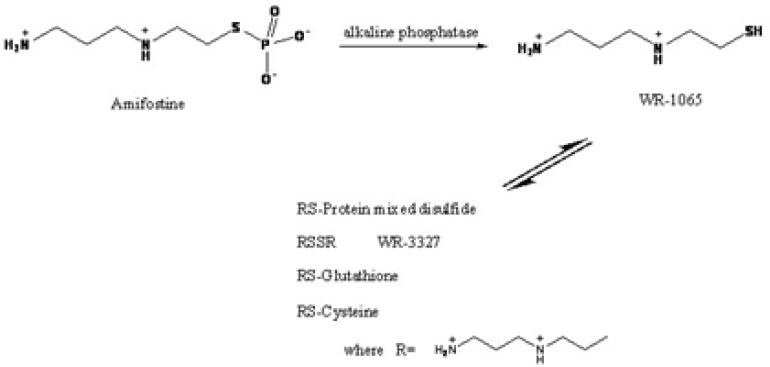
Structures and bioconversion pathway of amifostine and its metabolites

Amifostine (2-(3-aminopropylamino) ethylsulfanyl phosphonic acid, WR-2721) (AMF), a synthetic aminothiol compound, is used as a cytoprotective agent in cancer in radiotherapy and chemotherapy involving DNA-binding chemotherapeutic agents. As a prodrug, it isdephosphorylated in the tissue by alkaline phosphatase, to its active free thiol metabolite, WR-1065 (2-(3-aminopropylaminoethanethiol) (Figure 1). WR-1065 is able to scavenge free radicals, to deplete oxygen and covalently bind to active metabolites of antineoplastic agents. The sulfur-hydrogen bond of WR-1065 can easily donate its hydrogen ion to radiation-induced free radicals and hydrated electrons that candamage DNA, thereby decreasing cell damage [1].

WR-1065 exists in human plasma in various forms; only trace amounts [~1%] are in reduced

(sulfydryl) form, the remaining part is oxidized and exists as various disulfides. About 50% of WR-1065 is bound to proteins in plasma (via disulfide bond), whereas the remaining part exists as free disulfides, mostly as WR- 33287[2] (Figure1).

There is no universal technique for determination of both AMF and its major metabolite WR-1065 in plasma. The most widely used methods are: (1)direct electrochemical detection after high-performance liquid chromatography (HPLC) [3-5], (2) pre–column derivatization and fluorimetric detection of WR-1065 with monobrombimane (MB) and maleimide reagent (thioGloTM3) which derivatize thiol group of WR-1065 [6-8].

**Figure 2:**
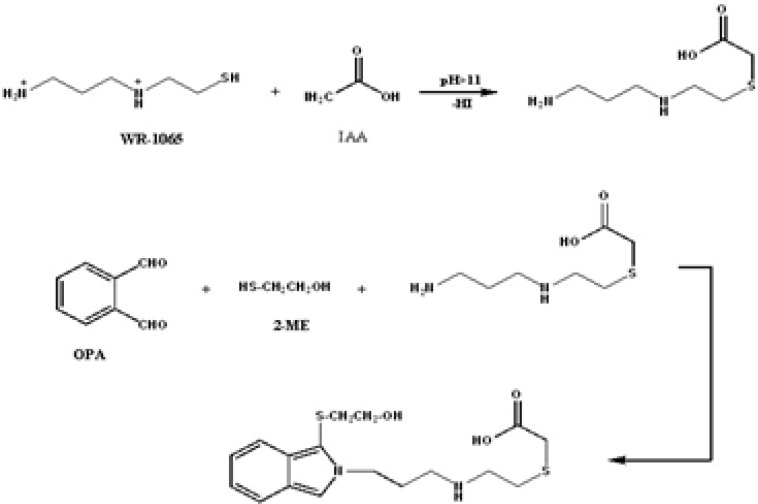
Alkylation of WR-1065 with IAA followed by derivatization with OPA-2-ME

Most of the reported methods require tedious extraction procedures, which are time-consuming, complex or both [11]. A method of sample pretreatment has been developed which reductively cleaves the sulfur-sulfur bond or WR-1065 disulfides, thus regenerating free WR-1065 [13]. The present study describes a rapid and sensitive and accurate HPLC method based on alkylation of free sulfydryl group with iodoacetic acid of AMF and/or its major metabolite, WR-1065, and derivatization of analytes with *o*-phthaldialdehyde (OPA) followed by UV detection (Figure 2). The method enables determination of the drug alone for *in-vitro* studies. Also, the method is applicable for simultaneously determination of AMF and its metabolite, with an acceptable accuracy at drug concentrations as low as 0.5 µgmL^-1 ^in plasma using a simple extraction procedure. The proposed assay method needs small sample volume (100 µL) [14-15].

Separation was performed on a reversed-phase Eurospher column and therefore, it allows easy optimization of the chromatographic conditions to obtain desirable resolution in a reasonable time, without interfering with endogenous plasma peaks. The sample preparation only involves a simple extraction procedure and no evaporation step is required.

It is necessary to mention that AMF has very short half-life (10 min) and rapidly convert to WR-1065. Thus, validation assessment of the proposed method was based on determination of the metabolite, WR-1065 [12].

## Experimental

*Chemicals*

AMF was supplied by ZiboPaxinPharm Chemical Company (China), dihydrochloride salt of WR-1065, OPA and 2-mercaptoethanol (2-ME) were obtained from Sigma- Aldrich company (France). L-cysteine, iodoaceticacid (IAA), HPLC-grade methanol,and all other chemicals were obtained from Merck (Darmstadt, Germany).Water was prepared by double distillation and purified additionally with a Millie-Q system.

*Chromatographic conditions*

HPLC system consisted of a model Manager 5000, a model Pump 1000, and a model D-14163 injector connected to a model PDA detector 2800, all from Knauer (Berlin, Germany). The separation was performed on Eurospher Performance (RP-18e, 100 × 4.6 mm) column from Knauer (Berlin, Germany).

An isocratic elution system was employed with mobile phase consisted of methanol and 0.03M phosphate buffer (40:60 v/v, respectively) adjusted to pH = 2.7 with a flow rate of 1.5 mLmin^−1^.

Chromatograms were monitored by diode array detection (200-400 nm) and displayed at single wavelength of 340 nm. The mobile phase was prepared daily and degassed by ultra-sonication before use. The mobile phase was not allowed to recirculate during the analysis.

*Standard solutions*

A stock solution (0.1 mgmL^−1^) of AMF was prepared in 0.05 M phosphate buffer pH = 6.4. Then, standard solutions were prepared at concentrations of 100, 90, 70, 50, 30, 10 and 1 µgmL^−1 ^by serial dilution. Also, a stock solution of WR-1065 were prepared in 0.05 M HCl solution.

Then, standard solutions were prepared at concentrations of 10, 50, 100, 200, 500, 1000 and 2000 µgmL^−1^ofWR-1065 in 0.05 M HCl solution and stored at -20°C until further use.

The internal standard (cysteine) solution was prepared by dissolving 3 mg cysteine in 10 mL 0.05 M HCl to obtain a concentration of 300µgmL^−1^ and stored at 4°C until further use.

*Plasma sample preparation*

To 100 μL of plasma in a polyethylene tube were added 20 μL of cysteine solution as internal standard (at a final concentration of 30 µgmL^−1^), 40 μL WR-1065 and 20µL 2-mercaptoethanol.

The sample was mixed and after incubation for 30 s at room temperature plasma proteins were removed by precipitation with 200 µL acetonitrile followed by centrifugation at 12000 g for 10 min. Then 40µL of supernatant was mixed with 100µL of IAA solution (0.8M in 0.1 M sodium borate buffer pH 10.5) and 240 µL of 0.1 M sodium borate buffer (pH 11.5). After incubation for 30 s at room temperature 40 µL of OPA-2-ME reagent was added and after 3 min 100 µL of reaction mixture was injected into the HPLC system.

*Stability*

The stability of WR-1065 was assessed for spiked plasma samples stored at −20°C for up to one week, and at ambient temperature for at least 24 h. The stability of each stock solution stored at 20°C was determined for up to 4 weeks by injecting appropriate dilution of the stock in 0.05HCl on day 1, 15 and 30 and comparing their peak areas with freshly prepared solution on the day of analysis. Samples were considered to be stable, if the assay values were within the acceptable limits of accuracy and precision.

*Plasma standard curve*

Blank plasma was prepared from heparinized whole-blood samples, collected from healthy volunteers and stored at 20°C. After thawing, 40 μL of one of the above-mentioned WR-1065 working standards were added to yield final concentrations of 1, 5, 20, 50, 100 and 200 µgmL^−1^. Internal standard solution was added to each of these samples to yield a concentration of 30 µgmL^−1^. The samples were then prepared for analysis as described above. Calibration curves were constructed by plotting peak area ratio (y) of WR-1065 to the internal standard versus WR-1065 concentrations (x). A linear regression method was used for quantitation.

*Precision and accuracy*

The precision and accuracy of the method were examined by adding known amounts of WR-1065 to pool plasma (quality control samples). For intra-day precision and accuracy, six replicate quality control samples at each concentration were assayed on the same day. The inter-day precision and accuracy were evaluated on three different days.

*Limit of quantification (LOQ) and recovery*

The analytical recovery for plasma at three different concentrations of WR-1065 (5, 50 and 200 µgmL^−1^) was determined. Known amounts of WR-1065 were added to drug-free plasma and the internal standard was then added. The relative recovery of WR-1065 was calculated by comparing the peak areas for extracted WR-1065 from spiked plasma and a standard solution of WR-1065 in 0.05 M HCl containing internal standard with the same initial concentration (six samples for each concentration level).

*Selectivity and specificity*

Control human plasma, obtained from three healthy volunteers, was assessed by the procedure as described above and compared with respective plasma samples to evaluate selectivity and specificity of the method.

## Results and Discussion

*Method development*

AMF and WR-1065 are primary amines and OPA is a suitable and selective derivatizing reagent for both compounds. Also, cysteine is structurally similar to WR-1065 (*i.e.* both are aminoacids) and reacts with OPA. But, WR-1065 and cysteine have sulfhydryl groups and therefore, they yield a weak response with OPA-2-ME reagent [16-17]. Thus, IAA was added to supernatant of plasma after deproteinization and before the addition of OPA–2-ME to prevent reformation of disulfide bonds of sulfhydryl group of the metabolite and the internal standard.

WR-1065 and cysteine exists in human plasma in varius forms. Only trace amounts are in reduced (sulfydryl) form. The remaining parts are oxidized and exist as various disulfides [18]. Therefore, it is necessary to use a reducing agent to release WR-1065. For this purpose 2-ME was added to plasma sample before protein precipitation. But 2-ME also possesses a SH group that can react with IAA and deactivates it. Thus, IAA concentration must exceed 2-ME concentration. In this research the quantity of IAA was four-times more than the quantity of 2-ME in the sample [13].

The optimum pH for derivatization of many endogenous amino acids lies in the range between 9 and 9.5 [19, 20]. Thus, a higher pH value used in the proposed method (*i.e*. pH = 11.5) enhances selectivity of the method by reducing derivatization of the endogenous amino acids.

**Figure 3:**
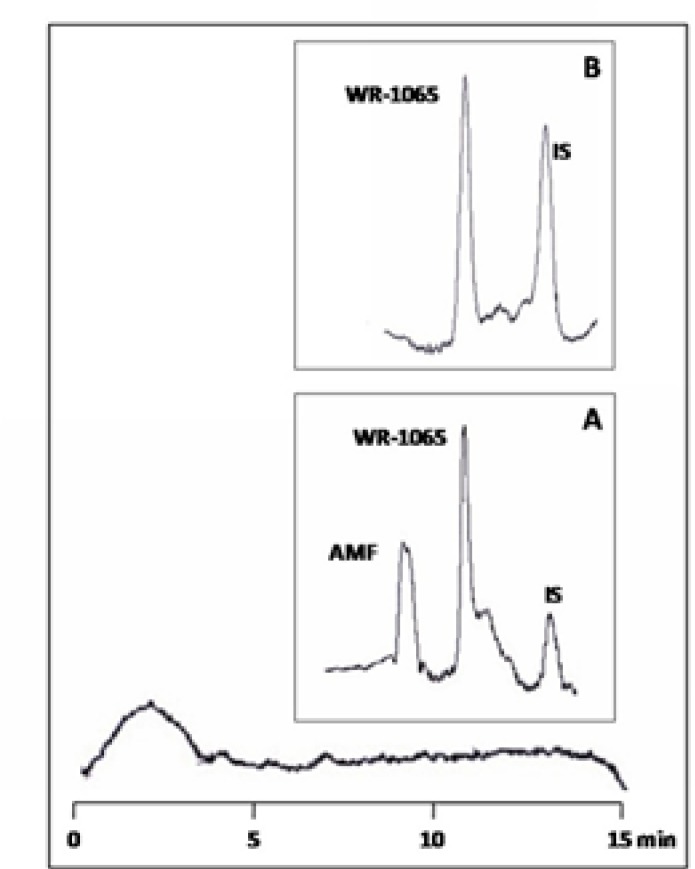
Chromatograms of blank plasma reacted with OPA/2-ME reagent (larger box); blank plasma spiked with 30 µgmL^−1 ^AMF, 50µgmL^−1^ WR-1065 and 30 µgmL^−1^L-cysteine (as internal standard, IS) (box A); and blank plasma spiked with 50µgmL^−1^ WR-1065 and 30 µgmL^−1^L-cysteine (box B).

Under the chromatographic conditions described, WR-1065 and AMF and the internal standard peaks were well resolved and no interfering peaks from endogenous plasma components were observed. A UV-VI is diode array detector was used for identification and examination of purity of the peaks. Figure 3 shows typical chromatograms of a plasma sample spiked with AMF and WR-1065 along with the internal standard and a blank plasma sample spiked with OPA/2-ME.

Retention times of WR-1065, AMF and cysteine were 8.2, 10.5 and 13.9 min, respectively. The formation of the OPA/thiol derivatives of WR-1065 and cysteine in the presence of IAA were simple and rapid reactions.

*Validation of the method*

A linear relationship between the absorbance at 340 nm and the concentration of WR-1065 was established over the examined concentration range (1-200 µgmL).

The average regression equation was found by applying the least squares method and calculated as Y = 0.0211X. The relative standard deviation for the slope was 1.1%and the average correlation coefficient was 0.993.

The repeatability of derivatization reaction was evaluated by calculating relative standard deviation (RSD) values of six consequtive injections of the working standard solutions. The RSD of peak area of WR-1065 was less than 10%.

For the sample preparation, several tedious extraction methods have been used for analysis of WR-1065 in biological fluids [5–9]. In addition, some of the methods involved using large volumes of plasma samples or toxic extraction agents which limited their application to analyze large numbers of clinical samples [3-5]. In our method, sample preparation involves simple extraction procedure and no evaporation step is required.

The analytical recovery for plasma at three different concentrations of WR-1065 was determined. Known amounts of WR-1065 were added to drug-free plasma in concentrations ranging from1–200 µgmL−1. The internal standard was added and the absolute recovery of WR-1065 was calculated by comparing the peak areas for extracted WR-1065 from spiked plasma and a standard solution of WR-1065 containing internal standard with the same initial concentration.

As shown in Table 1 the average recovery ofWR-1065 from plasma, determined at three different concentrations of the metabolite (5, 50, 200 µgmL−1), was 90 ± 0.9% (n = 6).

**Table 1:** Assay validation of WR-1065 in human plasma

**Spiked conc.** **(µg/ml)**	**Between-day assay (n=6)**			**Within-day assay (n=6)**		
	Mean	SD	RSD (%)	Mean	SD	RSD (%)
**5 **	5.9	0.5	8.5	5.4	0.9	16.7
**50**	49.8	4.5	11	52.4	5.6	10.7
**200**	217.2	21.7	10.0	217.2	26.6	12.2

Using OPA dervatization and UV detection, the limit of quantification (LOQ), as previously defined, was 0.5 µgmL^−1^ for WR-1065, which is sensitive enough for drug monitoring and for pharmacokinetic studies.

We assessed the precision of the method by repeated analysis of plasma specimens spiked with known amounts of WR-1065. As shown in Table 1, coefficients of variation were less than 10% which is acceptable for the routine measurement of WR-1065. Stability was determined for spiked plasma samples under the conditions aspreviously described. The results showed that the samples were stable during the applied conditions. The proposed method is well suited for routine application in the clinical laboratories because of the speed of analysis and simple extraction procedure. No change in the column efficiency and back pressure was also observed over the entire study time, proving its suitability.

## Conclusion

A robust and sensitive HPLC method has been described for analysis of WR-1065, as an active and major metabolite of amifostine, in human plasma. Derivatization with OPA is an efficient method for enhancing chromatographic detection of amifostine and other structurally related compounds such as WR-1065. In addition, the use of a simple procedure for sample preparation makes the proposed method a fast and reliable one for pharmacokinetic studies of WR-1065in humans.
